# Hospital and Institutional News

**Published:** 1913-12-20

**Authors:** 


### HOSPITAL AND INSTITUTIONAL NEWS

### THE QUEEN AT DERBY INFIRMARY.

During the King and Queen's visit to Chatsworth last week, her Majesty paid a private visit to the Derbyshire Royal Infirmary. A children's stand for 5,000 youngsters was erected outside the institution, where the Queen arrived about twelve. Mr. G. Herbert Strutt, chairman of the weekly board, was presented at the entrance, and in turn presented Colonel Buchanan, chairman of the house committee, Mr. Edmund Forster, secretary-superintendent, Dr. Vaudrey, senior physician, Mr. R. H. Luce, senior surgeon, Mr. E. Collier Green, senior member of the hon. medical staff, and Miss M. E. Sutcliffe, the matron. The Queen then entered the men's surgical ward, the women's surgical ward, and finally the children's ward, where there were 29 patients. Mr. H. T. Hicks, the hospital's gynæcologist, was sent for to explain the working of an incubator in which a four-weeks-old baby was being reared. The Queen then returned to the entrance hall, where she signed the visitors' book, in which Mr. Strutt drew her attention to records of previous royal visits in 1872, 1894, when King Edward, as Prince of Wales, with Queen Alexandra; and later Queen Victoria had visited the institution. Altogether the Queen spent half an hour in the institution. We may recall that the hospital, which consists of some dozen or more blocks, and contains 256 beds, was formally opened after rebuilding in 1894. Recent additions, however, include an eye department, the children's ward, given by Mr. Leslie Wright, a nurses' home, and a Finsen Light department. At the present time a second operating theatre is contemplated. This royal visit therefore has honoured an institution which has grown very largely indeed of late years.

### ST. GEORGE'S HOSPITAL.

The special meeting of the governors at the hospital on Friday, 19th inst., should be attended by a majority of the governors at least. The requisitionists urged in their circular letter "that the proposals of the house committee to create a special highly paid post for the treasurer, coupled with a request to their secretary to resign his post after 27½ years' service in the hospital, are contrary to the best interests of the hospital, and as such should be refused. As this new post will entail considerable extra expenditure, which is not justifiable in the present condition of the finances of the hospital, the undersigned protest against passing these proposals of the house committee." It is currently reported that the proposals in question are mainly supported by the medical staff, but we hesitate to credit the truth of this statement. They can scarcely be so blind as to work for the destruction of their own hospital, which these proposals if carried may in fact cause.

### "TRUTH" DOLL EXHIBITION.

For thirty-four years *Truth* has organised the dressing of dolls and their distribution with toys to the children throughout the Poor-Law institutions of the Metropolis. This year 32,500 dolls were exhibited at the Albert Hall on the 16th inst., when the Lord Mayor opened *Truth's* Show. The Editor, Mr. R. A. Bennett, who has the burden of this great exhibition on his hands every year, gave an interesting account of the history of the *Truth* dolls, and explained how the work had grown from 16,000 to 32,500 dolls in thirty years. This year's exhibition, and we have seen many, is probably the best *Truth* has ever held. The arranging and grouping of the dolls, their quality, and the excellence and taste of the dresses and tableaux, constitute the best doll show which London has ever seen. It was a pleasure to visit it, and anyone with half-an-hour to spare found much interest and instruction in examining the exhibits. Queen Mary's beautiful dolls are most attractive, artistic, and perfectly grouped. *Truth* is doing a public service in acting as almoner to all of us who are outside the workhouses but are desirous of giving the children within them as happy a Christmas as their circumstances permit. We hope many of our readers will send something by way of contributions in money to the *Truth* Toy Fund before the year is out. The four Saints exhibited are a masterpiece of artistic workmanship and finish. The Lady Mayoress won the sympathy of all present by her gracious words in opening the *Truth* Doll Show, and the members present constituted a record. Mr. Bennett and his co-workers deserve honour and the grateful thanks of all Londoners.

### A PENSION FUND FOR HOSPITAL OFFICERS.

This subject was the text of the speech made by Mr. Herbert Jennings, president of the Incorporated Association of Hospital Officers, at its annual dinner last week. He said that a Hospital Officers' Benevolent Fund had now been fairly launched, and referred to two recent instances in which members had come to the aid of their colleagues. The chief point in the speech was the statement that King Edward's Hospital Fund was sympathetically considering a scheme for a pension fund for hospital officers, so he thought that this deferred hope, for which many attempts had failed to produce a satisfactory scheme, might yet be realised. Mr. G. H. Hamilton, in proposing "The Visitors," referred to the value of lawyers and judges on hospital boards. In replying to this toast Lord Justice Kennedy, who has had considerable experience of work on a hospital board, strongly advocated the voluntary system, and referred to the difficulties in which taxation was involving charities, and to the possibility of receiving assistance from paying patients. In any case, he said, they wanted to keep the hospitals the homes of the sick poor. Mr. Jennings certainly made the most of his opportunity in raising the question of a pension fund, and we could have wished that there had been occasion for a discussion of the subject following his remarks. The need for hospital officers to place themselves upon a proper footing in this respect is not quite parallel to that which led to the formation of a pension fund for nurses, but it exists, and is of great importance. The difficulties in the way are varied, and the proposals numerous and conflicting. We should be glad to test hospital opinion on this subject, and therefore throw open our correspondence columns to a discussion of the desirability and means of establishing a pension fund for hospital officers.

### "STARVED TO DEATH" IN A SANATORIUM.

The Stourbridge and Halesowen Hospital Committee, at a meeting last week, presided over by Mr. J. Wooldridge, considered various allegations that have been made in the Lye district concerning the treatment of tuberculosis patients at the hospital. According to Mr. A. H. Gordon it was actually stated that a patient had been starved to death. The man, who had died since leaving the institution, Mr. Gordon had interviewed himself and investigated his case. The patient, he said, made no complaint against his treatment, but he asked Dr. E. W. Hardwicke, the medical superintendent, to refute the allegations. This Dr. Hardwicke did by giving a list of the food provided, and by stating that the man made no complaint on leaving the institution, but had criticised the open-air treatment, and said that the hospital was too cold. Mr. Wooldridge said he was convinced that the allegations against the staff were unfounded. The committee concurred, but passed no resolution, an omission which may very easily deprive their opinion of its official air so necessary when allegations are to be publicly repudiated.

### A WINDFALL FOR THE LONDON HOSPITAL.

A magnificent offer has been received by Mr. Sydney Holland in response to his appeal, noted by us last week, for the £30,000 needed to bring up the total to the sum of £100,000. The Commercial Union Assurance Company, Limited, as trustees of the late Sir William Dunn's will, and in the exercise of the discretionary powers entrusted to them for dealing with the residuary estate, have written to Mr. Holland undertaking to provide £15,000 on condition that a similar amount be subscribed by January 31. With such a magnificent send-off this last effort to make the quinquennial appeal a success should be assured of a speedy triumph, and if only the smaller contributors will seize the opportunity afforded them, the second £15,000 should be forthcoming before the Christmas season is over. The raising of this money will be the best Christmas and New Year present that the London Hospital could have, and by the generous action of the Commercial Union Assurance Company every giver can feel that his subscription is doubled. It must also be remembered that the system of quinquennial appeals is an attempt to make voluntary hospitals as free from excessive begging as voluntary charity can ever be.

### THE LIVERPOOL TUBERCULOSIS SCHEME.

Thanks to the co-operation of the Liverpool Insurance Committee and the City Council, the former is able to submit an important scheme for the treatment of all classes of tuberculosis in the City to the Insurance Commissioners. The financing of the scheme consists of an income of £40,000, made up by £10,000 from the Insurance Committee and £15,000 apiece from the City Council and the Government. The City contribution, it is pointed out, would mean only a halfpenny rate, as it already votes £7,500 a year for tuberculosis treatment. The promoters of the scheme then make the bold calculation that with this income of £40,000 proper treatment could be provided for every tuberculosis patient in the City of Liverpool. Institutional treatment would be provided by an extension of the Fazakerley Hospital, and a sanatorium for children suffering from tuberculosis of the joints is being built on the Leasowe Embankment by the Mersey. Dispensaries will supplement the work, in the varied way that was described in the article published on their theory and practice in The Hospital last week. So far as we are aware this is the most comprehensive city scheme yet drawn up, for it aims at a completeness of which most others have not ventured to predict.

### A CLEARING HOSPITAL COMPLETE.

The British Red Cross Society has very wisely given official recognition to a most useful sixpenny pamphlet intended for the guidance of Voluntary Aid Detachments of the Territorial Force. This is a "plan of a clearing hospital of 100 beds" with complete inventory of everything needed, by E. M. M., and published by Simpkin & Co. The plan itself is clear and simple; though susceptible of improvements, it is an intelligent attempt at a solution of the problem which will confront commanders of V.A.D.'s, if ever the Territorial Force is mobilised to resist invasion. In order to stand the wear and tear of camp life it should be mounted on linen, though we are not suggesting that the publishers should have done this at the exceedingly modest price asked for the pamphlet. The inventory of stores, requisites, and appliances of every kind is very sound and complete. Instruments and suture materials are not included, but are left to the discretion of the medical officer. The quantities of dressings suggested are minimal, and this part of the inventory could be expanded with advantage. When it is remembered that the War Office makes no attempt to supply these essential parts of any scheme of national defence, but expects County Associations to raise money for them by voluntary subscriptions amongst those who already offer their time and labour for nothing, this pamphlet can only be regarded as a useful eye-opener of official pretence and neglect. It should be of enormous value to commanders of Voluntary Aid Detachments.

### THE SURREY COUNTY HOSPITAL'S JUBILEE.

Two very definite and cogent reasons have been given by Mr. F. F. Smallpiece, chairman of the Royal Surrey County Hospital, for the financial difficulties which have led to the issue of the Jubilee appeal. Three years ago it was decided to open a ward, the Edward Ward, which had remained closed for lack of funds, and this raised the number of beds to 100, and increased the cost of maintenance by £600 a year. That step could not have been taken had not a fund been collected to allow of its opening, but in three years this fund has become exhausted. Secondly, the hospital's convalescent home at Worthing, though partly endowed by its founder, was still a charge upon the hospital's funds to the extent of £300 a year. It is thus clear that the current deficiency of £1,200 on the year's working is the result of the increased usefulness and efficiency of the hospital, and as such should appeal to the sense and generosity of Surrey, which, as among the wealthier counties of England, should be able to increase the annual subscriptions by £1,200, without devoting the Jubilee Fund to present expenses at all. Mr. Smallpiece quoted Sir Henry Burdett's written comment in the visitor's book that, "taken as a whole, this hospital has advanced by leaps and bounds in recent years"; and we hope that the general evidence of valuable work laid before the governors by Mr. Smallpiece will lead to the subscription of a sum worthy of the hospital, which has received a good start of something like £4,000 from a small group of its wealthier local supporters.

### MODERN HOSPITAL MUSICAL SOCIETIES.

The amateur in art is usually, and generally fairly, laughed out of court by the genuine artist, who feels that his work is too important and exacting to be taken up in anyone else's spare time. There is a noteworthy exception to this rule, however, for the amateur musician has achieved wonders in music both as an executant and as an educator of the public taste; indeed, it is hardly too much to say that the latter-day popularity of the Queen's Hall concert is the result of the amateur enthusiasm of years ago. While the amateur actor as a class has made "amateur theatricals" a bad joke, and certainly done nothing for the serious art of the drama, the amateur musician has set before himself the highest standard and insisted on the public taking music in the spirit of the master musicians. The latest instance of this is the Guy's Hospital Musical Society, whose concert last week in the institution comprised a programme which, in the words of the musical critic of the *Times*, "contained nothing that was not worth listening to and very little that did not receive a finished performance." Imagine Mr. A. B. Walkley, the dramatic critic of the same newspaper, being able to say that of any amateur dramatic performance, and you will understand the heights and depths to which the amateur in art may rise or fall. This society is formed by medical students and nurses, conducted at present by Mr. Denis Browne, with a string orchestra of a dozen or more players and a chorus of some sixty. Bach, Brahms, Purcell, and Grieg were all on the programme, to say nothing of Percy Grainger, while Mr. Clive Carey, the society's past conductor, took part in one of his own compositions for the piano and violin, and himself sang twice. No wonder the above-mentioned critic exclaimed, "The real interest (of the concert) lay in the fact that it was possible for a first-rate programme of this sort to be undertaken by a hospital and carried through by a number of very busy people in an entirely musical spirit." Let other hospital musical societies seek to earn similar expert praise.

### OUT-PATIENT RESTAURANTS.

Among the larger hospitals which have done something to secure the comfort of out-patients during the long wait that often attends their visits after perhaps a lengthy journey, by providing refreshments at cost price, the General Hospital, Birmingham, is now to be counted. These refreshment bars, or stalls, however, are not as common, nor as adequately managed as they ought to be, and the big hospitals have here, as in other respects, an example to set, and a standard of efficiency to maintain. Every out-patient waiting hall of any size should be provided with an adequate bar, and even where the attendance does not seem to warrant this some means should be afforded patients of gaining refreshments. The test of the necessity of having such a bar is whether or not the patients have to wait several hours before they are attended, and equally whether they generally come from long distances. Where either is the rule—and how often is it not?—a refreshment bar should be established.

### L.C.C. AND NURSING HOMES: PARLIAMENTARY BILL.

The Bill embodying the proposals of the London County Council with regard to the control of nursing homes is now being drawn up for presentation to Parliament. The Parliamentary Committee of the Council states that the object is to prevent immorality. Registration is to be made compulsory, but the Home Office consider the suggested fee for registration—namely, two guineas—too much, and the committee has therefore limited it to one guinea. Registration may be suspended or cancelled if the character of the person is deemed unsatisfactory on other grounds, or if the Act's provisions are contravened, but an appeal to a magistrate is allowed. If the Council believes any premises in the county, even if advertised as used for purposes to which these provisions do not apply, to be used as a disorderly house, it may authorise its officers to inspect them. Any information thus obtained will be forwarded to the authorities who usually take proceedings under the Disorderly Houses Acts. Lying-in homes are not included in this part of the Act, but in the part devoted to them registration at a fee not exceeding 10s. 6d. is enforced, the Council being the authority. Registration may be withheld if the premises or the person be deemed unsuitable. Before making an order to refuse to register or cancel or suspend registration, notice is to be given, and right of appeal to a magistrate allowed. In regard to nursing homes, the suitability of equipment of premises was not dealt with. The Parliamentary Committee report that they have included a power of the Council to make by-laws for safeguarding the health of women received in lying-in homes and for preventing immorality in connection with them. The Council's resolution of October 21 last was silent on this point. A penalty not exceeding £50 is provided for contravention of the provisions of the Act. The Council, subject to its regulations, are to have the right of entry into premises which there is reasonable cause to believe are being used for a lying-in home. The provision with regard to advertisements—that penalties attach to the publication of such after notice that registration has been refused, suspended, or cancelled—seems to the Parliamentary Committee to have equal application to nursing homes and lying-in homes, though it was not included in the Council's resolutions relating to the latter. No institutions are to be exempted from the Act by name, but exemption applies to those controlled by a Government department or any authority constituted by Parliament, lying-in homes where no payment is received, and any premises which the Council may specify by resolution. An appeal is provided to the Secretary of State against the refusal of the Council to exempt, which was not specifically provided for in the Council's resolution. The Lying-in Hospitals Act of 1773 is not repealed. The scope of the Bill here indicated may be usefully compared with the summary that appeared in The Hospital of November 15.

### LONDON'S LATEST PAY HOSPITAL.

The much-advertised Empire Hospital for Paying Patients in Vincent Square was open for inspection on Monday last previous to the admission of patients. Designed to cater for persons of moderate means who cannot afford the usual fees of a nursing home, the terms are from three to ten guineas a week. There are two theatres, for major and minor operations, and also a room for operations on septic cases. In addition to the customary medical and surgical, obstetrical cases are also received, and patients are attended by their own doctors. The equipment includes electric installation for baths and *x*-ray examinations and so on, while massage also may be carried out. The new nursing home has at least the advantage of being built and not adapted for its present purpose, but the fees asked, if £3 be indeed the lowest decided on, make little of its pretensions to cater for those who cannot afford an expensive nursing home. For this large class, represented practically by a payment of two guineas a week, there is still no recognised institution for both sexes in all non-infectious diseases, and the Empire Hospital will not help them. A descriptive account of the new institution appears elsewhere in this issue.

### SOUTHWARK GUARDIANS AND GUY'S HOSPITAL: A SUGGESTION.

There is a possibility of ending the long struggle between the Southwark Board of Guardians and Guy's Hospital, which has caused so much bitterness and friction. The hospital is situated in the area of the Guardians, but the infirmary is over three miles away. When patients at Guy's Hospital are referred to the Guardians they have to make this long journey, with the result that some painful incidents have arisen. Now, however, the Bermondsey Board of Guardians have offered to take patients into their infirmary, and, as the Bermondsey institution is much nearer to Guy's, it has been suggested that Southwark people attending Guy's should be sent by the Guy's authorities to the Bermondsey Infirmary. If this is adopted it will meet the suggestions which have been pressed upon the Southwark Guardians to equip an emergency station close to Guy's Hospital for urgent cases. Mr. J. O. Devereux, the Chairman of the Southwark Guardians, is strongly in favour of the scheme, and it is to be hoped that he will induce his colleagues to accept the offer pending a better solution of the difficulty.

### CENTRAL ASSOCIATION FOR THE MENTALLY DEFECTIVE.

The Provisional Council formed by representatives of voluntary agencies to organise the Central Association for the Care of the Mentally Defective has received official benediction. The Board of Control, in the person of its Chairman, Sir William Byrne, has expressed its approval, and the local authorities have now to express satisfaction in their turn. The composition of the Provisional Council is to include all the interests ultimately to be represented on the Central Association, and an executive committee of thirty-six is being formed. The Central Association should have a most useful and strenuous future before it, for if the Act is really to be in force on the scale contemplated by its promoters, State action, unaided by voluntary effort, would quickly be paralysed by the enormous expense involved in dealing with the numbers that will require attention and treatment. In fact, as we have seen in the case of consumption, once an organised effort to combat any particular defect or disease is made, the number of sufferers leaps up alarmingly, through the very success of efforts which reveal early cases that previously would never have been recognised at that stage at all.

### HAMPSTEAD'S NEW HEALTH INSTITUTE.

A somewhat novel form has been given to King Edward Memorial institutions by the opening of the Hampstead Health Institute, the aim of which is to allow the voluntary and municipal health agencies of the borough to work together and be housed under one roof. Under the name of Kingsgate House the new institution has now been opened, and consists of a basement devoted to the School of Domestic Economy for Girls and Mothers; a ground floor where the borough tuberculosis dispensary and a bacteriological laboratory are placed; while on the first floor is the children's clinic of the Council of Social Welfare. There is also a room for minor operations performed by the medical staff of the Kilburn Provident Medical Institute and the Hampstead Provident Dispensary; and an arrangement exists between the Council and the parents by which the children of the two provident dispensaries are under medical inspection by their own doctor up to school age, when the London County Council school doctor becomes responsible for it. There is also a social room, in which first-aid classes, lectures, and meetings will be held. As far as we are aware, this attempt to combine voluntary and municipal health work in one centre is original, and is certainly a noteworthy example of the public spirit of all concerned to secure co-operation and to prevent overlapping.

### ALTERNATIVES TO THE TENEMENT SYSTEM.

The appalling condition of the housing of the working-classes in Dublin which has been once more revealed, for it is an oft-repeated revelation, at the inquiry has led to the usual confusion in regard to remedies. Sir Charles Cameron, chief medical officer of health for Dublin, on being recalled, has said that he would like to see the tenement system cleared away, and in place of the impracticable schemes put forward of late, which would cost five or six million pounds, he still believed that the best class of tenement houses could be reconstructed and let as flats at rents varying from 6s. to 10s. a week. There would at all events be less loss on his scheme. That the Census revealed twelve persons in one room was explained by the fact that evicted families were received by their relatives; about 140 ejectments were heard in the two police courts every week. We confess that this explanation is hardly likely to lessen the indignation which Sir Charles says has been excited over this aspect of the case. The housing problem is not a problem of houses, but a problem of wages, and as long as a class exists which cannot afford to pay rent sufficient to cover a decent human habitation indecent overcrowding will inevitably occur, and inquiries and reforms will shift only its locality. This is the unpalatable truth, but until society recognises it there is something in the suggestion of Mr. Travers, chief of the sanitary staff, that tenements be abolished and the occupants be transferred to municipal lodging-houses and self-contained cottages. While no houses can be provided commercially for those who cannot afford to pay any rent, public bodies may be preferably equipped for dealing with the impossible problem of providing something for nothing.

### TOWN TREES AND PUBLIC HEALTH.

The Hon. Secretary of the Eastbourne Medical Society recently caused some perturbation in the Corporation by informing the Town Council that his Society has passed a resolution to the effect that the trees which line many of the roads in the borough are from their numbers and size detrimental to health. "It was a sinful thing," said Alderman Keay, "that it should go forth that they in Eastbourne were suffering from the trees." In protest he brought forward statements from the medical officer of health, stating that the town's death rate had fallen from fifteen to ten per 1,000 per annum in the last twenty years, that the humidity of Eastbourne compared favourably with other towns, but that the medical officer was in favour of the trees being thinned where necessary. Eastbourne exploits the dividend-holding population, and those who cater for them, to such an extent that it may well dread any falling off in popularity among classes so timid as sea-side visitors. But who or what induced the Medical Society to convert into a scare a simple matter of town gardening carelessly done?

### THE ORDER OF ST. JOHN.

A batch of new promotions and appointments in the Order of St. John of Jerusalem in England is announced. Eleven new knights of grace are upon the list, two of whom are promoted from honorary associate, and have presumably therefore rendered services to the Order or to the cause of hospitals in England. These two are Colonel W. H. Bull, a very well-known Territorial R.A.M.C. officer and highly respected practitioner in Buckinghamshire; and Mr. Harry Littlewood, whose record is unknown to us. Dr. Cluny Macpherson is one of the new knights; but the medical directories do not contain this name, and possibly he may be a doctor of some faculty other than medicine. None of the other knights or of the new esquires are prominent in the hospital world; so that the old principle of neglecting those who do the real work of the Order by taking part in the honorary management of voluntary hospitals or by labouring for the Ambulance Association still seems to be the guiding factor in the allotment of decorations. The medical profession is slighted consistently and regularly by the governors of the Order; but the institutional world is treated even worse still by the Hospitallers of St. John.

### HOSPITAL FOR BABIES.

The Government of New South Wales has determined upon a step that it is hoped will reduce the death-rate of infants in that State, writes our Australian correspondent. A Babies' Hospital has been established at Vaucluse, a suburb of Sydney, with accommodation at first for 40 sick babies from the age of one day to one year. Where necessary the mother will be admitted with her baby. Dr. Paton, chief Government medical officer, is to be in charge, assisted by a board of women directors. The intention is to combat the disease of gastro-enteritis, which in Australia as elsewhere carries off many infants in summer. The hospital was due for opening about the middle of last month, which is late for New South Wales' summer.

### THE CLAPHAM MATERNITY HOSPITAL.

An appeal is being issued on behalf of this institution, which needs £8,000 for reconstruction purposes recommended by King Edward VII.'s Hospital Fund, which has promised substantial help if the plans are carried out with the approval of the committee. The claims of this excellent and unassuming charity are in some danger of being overlooked, perhaps, owing to the prominence which has been given during the last year or two to the projected new women's hospital at Clapham. Briefly, the Clapham Maternity Hospital dates from 1889; contains fifty beds, ten of which are used as a waiting home for expectant mothers; and has a large out-patient district. Many of the best-known woman obstetricians in London have been connected with this charity, whose sphere of work affords splendid experience in midwifery. A lady has offered £50 if nine others will give similar sums before the end of this year.

### HOSPITAL OFFICERS' CONFLICTING OPINIONS.

Two curious inquests were held recently at Hull, and have given rise to comments in the local press as well as to difficult positions for the jury. In one case a man died in the Hull Royal Infirmary from what appears to have been some form of meningitis associated with an acute inflammation of the lungs. A few weeks previously he had suffered from a crushed toe which kept him off work for a few days, but had entirely healed before the fatal illness commenced. One professional witness from the Infirmary expressed himself satisfied that death was due to septicæmia consequent upon the injury to the toe; but the patient's private practitioner, and also the pathologist who conducted the *post-mortem* examination, disagreed with this, and denied all connection between the two lesions. Eventually, after some hesitation, the jury accepted the latter view. The obvious flaw of the professional evidence, as it is reported in the *Hull Daily Mail*, was the omission of any bacteriological report on the meningitis. If the latter were streptococcal, the crushed toe might possibly have been the portal of entry; but if it was pneumococcal, or tuberculous, or diplococcal, this hypothesis would be discredited. In an institution of the size and standing of this one, such an investigation, in an inquest case especially, should surely have been carried out. In the second case a doctor is reported to have made dogmatic statements concerning a man who died of tuberculosis which are certainly not justified by the present state of scientific knowledge. A sympathetic jury brought in a verdict of accidental death, which, on the face of matters, was probably quite unjustifiable and erroneous.

### THE INSURANCE OF RADIUM.

According to the daily press, insurance policies have lately been effected by various hospitals upon quantities of radium of the aggregate value of about £20,000. The policies, it is said, provide that the radium (or mesothorium, in some cases) shall be kept, when not in use, in hermetically sealed bottles or capsules placed in lead-lined safes. The risk of gradual deterioration is excluded by the terms of the policy, and also that of misappropriation by patients, of which it is rumoured that several cases have already occurred. It is obvious that the insurance of radium must present unusual difficulties, even to such experts as the Lloyd's agents and underwriters. Radium exists in such minute quantities that it would be an easily mislaid article, to say nothing of the fact that the identification of any particular lost piece would be practically impossible. Again, the adulteration of radium is not an unknown art; and the detection of such an act is a long, costly and difficult process. Notwithstanding these formidable obstacles, apparently a good many policies have been taken up; the rates are said to be between fifteen shillings and one pound per cent. per annum.

### OUR PRIZES AND THE SPECIAL APPEAL NUMBER.

We publish this week the special Christmas Appeal Number, which has additional interest from the fact that it contains the prize essays which were invited for the best descriptions of How to Spend Christmas in Hospital from the different points of view of the various sections of the staff. The names of the prize-winners and the amount of their prizes are to be found in an introductory note which precedes the essays, which also contain illustrations of great interest. It is believed that these first-hand descriptions of what Christmas means to the workers in hospital will bring home to the public, as no other method could do, the purpose and value of the voluntary hospital system. All within reach of hospital committees, who should do their best to circulate the Special Number, will turn from the essays themselves to the tabulated needs of the different institutions, and thereby find simply set forth the claims of each and all to their support. We earnestly hope that the efforts which we have made this year will make the Christmas appeal more living than ever to all classes of the public. The price of the Appeal Number is one penny.

### THIS WEEK'S DRUG MARKET.

An otherwise dull week in the drug market has been relieved by the announcement of the makers of quinine of their decision to raise the price of the sulphate by three-halfpence per ounce, which is equivalent to an advance of rather over 12 per cent. Although an advance had been expected ever since the agreement between the growers of cinchona bark in Java and the manufacturers of quinine was signed in July last, the market had been so quiet recently that buyers had taken little interest in it, and the announcement came at a moment when it was least expected. It is thought by those who know the quinine market that further advances in price will take place in due course, and it is probable that no useful purpose would be served by refraining from making purchases. Menthol, which during the last few weeks has declined in value very considerably, shows signs of recovery, and the present might not be an unfavourable moment to cover requirements. Opium is practically unchanged; the advanced price of morphine is well maintained, and codeine is rather dearer. Cream of tartar still has a lower tendency, and the price of castor oil tends upwards. Cocaine is rather lower in price. The high value of citric acid is barely maintained in the absence of business in the article. Lower values for essence of lemon are anticipated. Cod-liver oil is practically unchanged in price, and very little business is being done.

